# (*E*)-1-(4,4′′-Difluoro-5′-meth­oxy-1,1′:3′,1′′-terphenyl-4′-yl)-3-(4-methyl­phen­yl)prop-2-en-1-one

**DOI:** 10.1107/S160053681104579X

**Published:** 2011-11-05

**Authors:** Richard Betz, Thomas Gerber, Eric Hosten, Seranthimata Samshuddin, Badiadka Narayana, Hemmige S. Yathirajan

**Affiliations:** a Nelson Mandela Metropolitan University, Summerstrand Campus, Department of Chemistry, University Way, Summerstrand, PO Box 77000, Port Elizabeth 6031, South Africa; b Mangalore University, Department of Studies in Chemistry, Mangalagangotri 574 199, India; c University of Mysore, Department of Studies in Chemistry, Manasagangotri, Mysore 570 006, India

## Abstract

In the *meta*-terphenyl fragment of the title mol­ecule, C_29_H_22_F_2_O_2_, the two fluoro­phenyl rings are twisted from the central benzene ring by 46.72 (6) and 41.70 (6)°, respectively. In the crystal, weak C—H⋯O and C—H⋯F hydrogen bonds link the mol­ecules into layers parallel to the *ab* plane. The crystal packing exhibits π–π inter­actions, the shortest distance between the centroids of aromatic rings being 3.6364 (7) Å.

## Related literature

For the pharmacological importance of terphenyls, see: Liu (2006 ▶). For our studies of different chalcone derivatives, see: Samshuddin *et al.* (2011**a* ▶,b* ▶); Fun *et al.* (2010**a* ▶,b* ▶); Jasinski *et al.* (2010**a* ▶,b* ▶); Baktir *et al.* (2011**a* ▶,b* ▶). For graph-set analysis of hydrogen bonds, see: Etter *et al.* (1990 ▶); Bernstein *et al.* (1995 ▶).
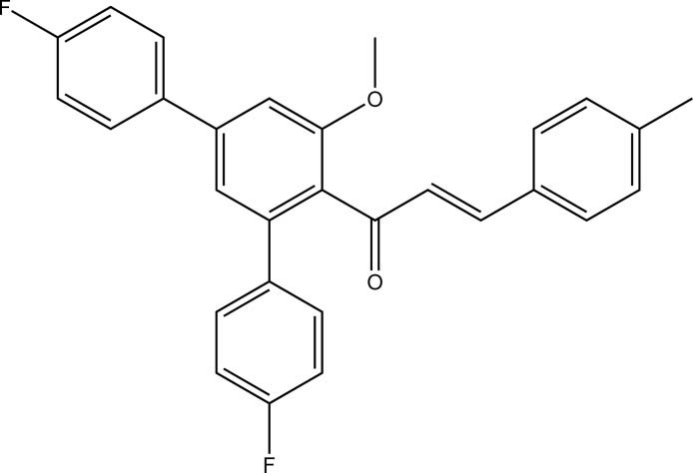


## Experimental

### 

#### Crystal data

C_29_H_22_F_2_O_2_
*M* *_r_* = 440.47
Triclinic, 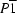

*a* = 6.9020 (3) Å
*b* = 11.3965 (6) Å
*c* = 14.8362 (8) Å
α = 96.177 (2)°
β = 93.381 (2)°
γ = 106.446 (2)°
*V* = 1107.85 (10) Å^3^
*Z* = 2
Mo *K*α radiation
μ = 0.09 mm^−1^
*T* = 200 K
0.36 × 0.24 × 0.11 mm

#### Data collection

Bruker APEXII CCD diffractometer
20122 measured reflections
5516 independent reflections
4020 reflections with *I* > 2σ(*I*)
*R*_int_ = 0.042

#### Refinement

*R*[*F* ^2^ > 2σ(*F* ^2^)] = 0.039
*wR*(*F* ^2^) = 0.109
*S* = 1.06
5516 reflections
300 parameters
H-atom parameters constrained
Δρ_max_ = 0.26 e Å^−3^
Δρ_min_ = −0.24 e Å^−3^

### 

Data collection: *APEX2* (Bruker, 2010 ▶); cell refinement: *SAINT* (Bruker, 2010 ▶); data reduction: *SAINT*; program(s) used to solve structure: *SHELXS97* (Sheldrick, 2008 ▶); program(s) used to refine structure: *SHELXL97* (Sheldrick, 2008 ▶); molecular graphics: *ORTEP-3* (Farrugia, 1997 ▶) and *Mercury* (Macrae *et al.*, 2008 ▶); software used to prepare material for publication: *SHELXL97* and *PLATON* (Spek, 2009 ▶).

## Supplementary Material

Crystal structure: contains datablock(s) I, global. DOI: 10.1107/S160053681104579X/cv5183sup1.cif

Supplementary material file. DOI: 10.1107/S160053681104579X/cv5183Isup2.cdx

Structure factors: contains datablock(s) I. DOI: 10.1107/S160053681104579X/cv5183Isup3.hkl

Supplementary material file. DOI: 10.1107/S160053681104579X/cv5183Isup4.cml

Additional supplementary materials:  crystallographic information; 3D view; checkCIF report

## Figures and Tables

**Table 1:** Hydrogen-bond geometry (Å, °)

*D*—H⋯*A*	*D*—H	H⋯*A*	*D*⋯*A*	*D*—H⋯*A*
C4—H4*B*⋯F1^i^	0.98	2.46	3.3756 (14)	156
C25—H25⋯O1^ii^	0.95	2.43	3.2812 (15)	149

Symmetry codes: (i) 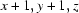; (ii) 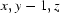.

**Fig. 1.**
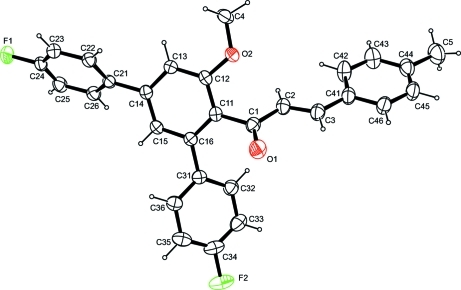
The molecular structure of (I) with atomic labels and anisotropic displacement ellipsoids (drawn at 50% probability level).

**Fig. 2.**
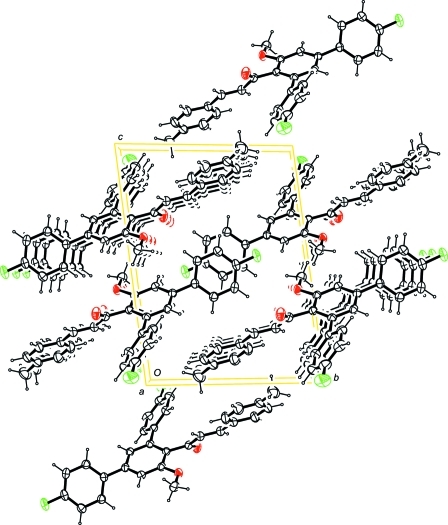
A portion of the crystal packing viewed down the a-axis.

## supplementary crystallographic information

### Comment

In view of pharmacological importance of terphenyls (Liu, 2006) and chalcones, and in continuation of our works on the synthesis of various derivatives of 4,4'-difluoro chalcone (Samshuddin *et al.*, 2011**a*,b*; Fun *et al.*, 2010**a*,b*; Jasinski *et al.*, 2010**a*,b*; Baktir *et al.*, 2011**a*,b*), the molecular and crystal structure of the title compound (I) is reported.

In (I) (Fig. 1), the C=C double of the Michael system is (*E*)-configured. The least-squares planes defined by the carbon atoms of the *para*-fluoro phenyl rings of the terphenyl moiety and its central phenyl ring enclose angles of 41.70 (6)° and 46.72 (6)°, respectively.

In the crystal structure, intermolecular C–H···O and C–H···F contacts are present (Table 1). While the C–H···O contacts are apparent between the ketonic oxygen atom and one of the phenyl-bonded hydrogen atoms, the C–H···F contacts are supported by one of the hydrogen atoms of the methoxy substituent on the terphenyl's central phenyl group. In terms of graph-set analysis (Etter *et al.*, 1990; Bernstein *et al.*, 1995), the C–H···O contacts necessitate a *C*^1^_1_(10) descriptor on the unitary level and the C–H···F contacts necessitate a *C*^1^_1_(11) descriptor on the same level. These two antidromic chains connect the molecules to planes perpendicular to the crystallographic *c* axis. The shortest intercentroid distance between two aromatic systems was found at 3.6364 (7) Å and is apparent between one of the *para*-fluoro phenyl moieties and its symmetry-generated equivalent. The packing of the title compound in the crystal structure is shown in Figure 2.

### Experimental

To a mixture of 1-(4,4''-difluoro-5'-methoxy-1,1':3',1''-terphenyl-4'-yl) ethanone (0.338 g, 0.001 mol) and 4-methylbenzaldehyde (0.120 g, 0.001 mol) in 30 ml e thanol, 1 ml of 10% sodium hydroxide solution was added and stirred at 278–283 K for 3 h. The precipitate formed was collected by filtration and purified by recrystallization from ethanol (yield: 76%). Single crystals suitable for the X-ray diffraction study were grown from DMF by slow evaporation at room temperature.

### Refinement

C-bound H atoms were placed in calculated positions (C—H 0.95 Å for aromatic and vinylic carbon atoms) and were included in the refinement in the riding model approximation, with *U*_iso_(H) set to 1.2*U*_eq_(C). The H atoms of the methyl groups were allowed to rotate with a fixed angle around the C—C bond to best fit the experimental electron density (HFIX 137 in the *SHELX* program suite (Sheldrick, 2008)), with *U*_iso_(H) set to 1.5*U*_eq_(C).

### Crystal data

**Table:**

C_29_H_22_F_2_O_2_	*Z* = 2
*M**_r_* = 440.47	*F*(000) = 460
Triclinic, *P*1	*D*_x_ = 1.320 Mg m^−^^3^
Hall symbol: -P 1	Melting point: 465 K
*a* = 6.9020 (3) Å	Mo *K*α radiation, λ = 0.71073 Å
*b* = 11.3965 (6) Å	Cell parameters from 8632 reflections
*c* = 14.8362 (8) Å	θ = 2.5–28.3°
α = 96.177 (2)°	µ = 0.09 mm^−^^1^
β = 93.381 (2)°	*T* = 200 K
γ = 106.446 (2)°	Platelet, colourless
*V* = 1107.85 (10) Å^3^	0.36 × 0.24 × 0.11 mm

### Data collection

**Table:**

Bruker APEXII CCD diffractometer	4020 reflections with *I* > 2σ(*I*)
Radiation source: fine-focus sealed tube	*R*_int_ = 0.042
graphite	θ_max_ = 28.4°, θ_min_ = 1.9°
φ and ω scans	*h* = −9→9
20122 measured reflections	*k* = −15→15
5516 independent reflections	*l* = −19→19

### Refinement

**Table:**

Refinement on *F*^2^	Primary atom site location: structure-invariant direct methods
Least-squares matrix: full	Secondary atom site location: difference Fourier map
*R*[*F*^2^ > 2σ(*F*^2^)] = 0.039	Hydrogen site location: inferred from neighbouring sites
*wR*(*F*^2^) = 0.109	H-atom parameters constrained
*S* = 1.06	*w* = 1/[σ^2^(*F*_o_^2^) + (0.0547*P*)^2^ + 0.0888*P*] where *P* = (*F*_o_^2^ + 2*F*_c_^2^)/3
5516 reflections	(Δ/σ)_max_ < 0.001
300 parameters	Δρ_max_ = 0.26 e Å^−^^3^
0 restraints	Δρ_min_ = −0.24 e Å^−^^3^

### Fractional atomic coordinates and isotropic or equivalent isotropic displacement parameters (Å^2^)

**Table:**

	*x*	*y*	*z*	*U*_iso_*/*U*_eq_	
F1	−0.32392 (11)	−0.70671 (6)	0.46700 (6)	0.0469 (2)	
F2	−0.80404 (14)	0.05047 (10)	0.94895 (6)	0.0661 (3)	
O1	−0.20131 (13)	0.21294 (8)	0.70190 (7)	0.0448 (2)	
O2	0.19220 (12)	0.09542 (7)	0.60606 (6)	0.0358 (2)	
C1	−0.06141 (17)	0.17004 (10)	0.71538 (8)	0.0305 (3)	
C2	0.13900 (18)	0.24573 (11)	0.75946 (8)	0.0336 (3)	
H2	0.2390	0.2061	0.7733	0.040*	
C3	0.18420 (19)	0.36734 (11)	0.78036 (9)	0.0364 (3)	
H3	0.0811	0.4041	0.7651	0.044*	
C4	0.34886 (17)	0.06610 (12)	0.55736 (9)	0.0376 (3)	
H4A	0.4098	0.0142	0.5913	0.056*	
H4B	0.4532	0.1424	0.5503	0.056*	
H4C	0.2909	0.0216	0.4972	0.056*	
C5	0.9333 (2)	0.70099 (15)	0.96324 (12)	0.0630 (5)	
H5A	0.9502	0.7847	0.9482	0.095*	
H5B	1.0489	0.6733	0.9450	0.095*	
H5C	0.9263	0.7008	1.0290	0.095*	
C11	−0.08873 (15)	0.03487 (10)	0.68885 (8)	0.0263 (2)	
C12	0.04172 (15)	0.00001 (10)	0.62946 (8)	0.0270 (2)	
C13	0.01112 (15)	−0.12245 (10)	0.59474 (8)	0.0273 (2)	
H13	0.0981	−0.1439	0.5528	0.033*	
C14	−0.14791 (15)	−0.21398 (10)	0.62168 (8)	0.0265 (2)	
C15	−0.27208 (15)	−0.18035 (10)	0.68396 (8)	0.0273 (2)	
H15	−0.3764	−0.2430	0.7045	0.033*	
C16	−0.24777 (15)	−0.05737 (10)	0.71708 (8)	0.0260 (2)	
C21	−0.18947 (15)	−0.34472 (10)	0.58167 (8)	0.0268 (2)	
C22	−0.19231 (16)	−0.37483 (11)	0.48819 (8)	0.0308 (3)	
H22	−0.1617	−0.3109	0.4505	0.037*	
C23	−0.23905 (16)	−0.49661 (11)	0.44927 (9)	0.0334 (3)	
H23	−0.2425	−0.5172	0.3853	0.040*	
C24	−0.28010 (16)	−0.58650 (10)	0.50542 (9)	0.0327 (3)	
C25	−0.27847 (17)	−0.56188 (11)	0.59813 (9)	0.0354 (3)	
H25	−0.3069	−0.6266	0.6351	0.042*	
C26	−0.23396 (17)	−0.43962 (11)	0.63608 (9)	0.0318 (3)	
H26	−0.2338	−0.4202	0.7000	0.038*	
C31	−0.39190 (16)	−0.02834 (10)	0.78062 (8)	0.0279 (2)	
C32	−0.32771 (18)	0.05884 (11)	0.85792 (8)	0.0351 (3)	
H32	−0.1872	0.1002	0.8720	0.042*	
C33	−0.4659 (2)	0.08601 (13)	0.91445 (9)	0.0427 (3)	
H33	−0.4220	0.1466	0.9664	0.051*	
C34	−0.6676 (2)	0.02359 (13)	0.89374 (10)	0.0425 (3)	
C35	−0.73750 (18)	−0.06496 (13)	0.82011 (9)	0.0395 (3)	
H35	−0.8780	−0.1077	0.8081	0.047*	
C36	−0.59796 (16)	−0.09080 (11)	0.76341 (8)	0.0325 (3)	
H36	−0.6438	−0.1521	0.7120	0.039*	
C41	0.3765 (2)	0.45038 (11)	0.82446 (9)	0.0373 (3)	
C42	0.5431 (2)	0.40945 (13)	0.84736 (11)	0.0496 (4)	
H42	0.5346	0.3248	0.8328	0.059*	
C43	0.7201 (2)	0.49057 (13)	0.89099 (11)	0.0526 (4)	
H43	0.8312	0.4604	0.9061	0.063*	
C44	0.7401 (2)	0.61507 (12)	0.91337 (9)	0.0456 (3)	
C45	0.5773 (2)	0.65663 (12)	0.88825 (10)	0.0477 (4)	
H45	0.5884	0.7419	0.9011	0.057*	
C46	0.3984 (2)	0.57627 (12)	0.84473 (10)	0.0435 (3)	
H46	0.2889	0.6073	0.8284	0.052*	

### Atomic displacement parameters (Å^2^)

**Table:**

	*U*^11^	*U*^22^	*U*^33^	*U*^12^	*U*^13^	*U*^23^
F1	0.0460 (4)	0.0236 (4)	0.0651 (6)	0.0042 (3)	0.0076 (4)	−0.0058 (3)
F2	0.0647 (6)	0.0905 (7)	0.0592 (6)	0.0438 (5)	0.0305 (5)	0.0092 (5)
O1	0.0439 (5)	0.0328 (5)	0.0615 (7)	0.0168 (4)	0.0003 (4)	0.0101 (4)
O2	0.0357 (4)	0.0264 (4)	0.0418 (5)	0.0010 (3)	0.0141 (4)	0.0051 (4)
C1	0.0366 (6)	0.0260 (6)	0.0301 (6)	0.0095 (5)	0.0056 (5)	0.0066 (5)
C2	0.0384 (6)	0.0270 (6)	0.0340 (7)	0.0075 (5)	0.0025 (5)	0.0041 (5)
C3	0.0450 (7)	0.0280 (6)	0.0352 (7)	0.0087 (5)	0.0063 (5)	0.0038 (5)
C4	0.0288 (6)	0.0398 (7)	0.0415 (7)	0.0032 (5)	0.0100 (5)	0.0085 (6)
C5	0.0609 (9)	0.0533 (10)	0.0569 (10)	−0.0055 (7)	0.0006 (8)	−0.0081 (8)
C11	0.0274 (5)	0.0243 (5)	0.0264 (6)	0.0067 (4)	−0.0007 (4)	0.0039 (4)
C12	0.0253 (5)	0.0251 (5)	0.0288 (6)	0.0036 (4)	0.0011 (4)	0.0059 (4)
C13	0.0249 (5)	0.0274 (6)	0.0294 (6)	0.0071 (4)	0.0036 (4)	0.0030 (5)
C14	0.0258 (5)	0.0245 (5)	0.0284 (6)	0.0067 (4)	−0.0003 (4)	0.0040 (4)
C15	0.0247 (5)	0.0255 (6)	0.0301 (6)	0.0039 (4)	0.0029 (4)	0.0059 (5)
C16	0.0251 (5)	0.0273 (6)	0.0256 (6)	0.0077 (4)	0.0005 (4)	0.0040 (4)
C21	0.0222 (5)	0.0240 (5)	0.0335 (6)	0.0059 (4)	0.0029 (4)	0.0033 (4)
C22	0.0298 (5)	0.0274 (6)	0.0345 (7)	0.0067 (4)	0.0048 (5)	0.0047 (5)
C23	0.0296 (5)	0.0320 (6)	0.0359 (7)	0.0068 (5)	0.0035 (5)	−0.0015 (5)
C24	0.0248 (5)	0.0224 (6)	0.0481 (8)	0.0049 (4)	0.0034 (5)	−0.0023 (5)
C25	0.0339 (6)	0.0248 (6)	0.0479 (8)	0.0067 (5)	0.0060 (5)	0.0099 (5)
C26	0.0314 (5)	0.0300 (6)	0.0336 (6)	0.0079 (4)	0.0043 (5)	0.0051 (5)
C31	0.0298 (5)	0.0287 (6)	0.0283 (6)	0.0116 (4)	0.0044 (4)	0.0078 (5)
C32	0.0375 (6)	0.0340 (7)	0.0334 (7)	0.0101 (5)	0.0041 (5)	0.0032 (5)
C33	0.0552 (8)	0.0406 (7)	0.0353 (7)	0.0188 (6)	0.0097 (6)	0.0017 (6)
C34	0.0476 (7)	0.0536 (8)	0.0394 (8)	0.0298 (6)	0.0190 (6)	0.0143 (6)
C35	0.0302 (6)	0.0520 (8)	0.0424 (8)	0.0173 (5)	0.0078 (5)	0.0164 (6)
C36	0.0306 (5)	0.0362 (6)	0.0326 (6)	0.0118 (5)	0.0026 (5)	0.0077 (5)
C41	0.0484 (7)	0.0263 (6)	0.0334 (7)	0.0046 (5)	0.0065 (5)	0.0020 (5)
C42	0.0581 (8)	0.0282 (7)	0.0574 (9)	0.0076 (6)	−0.0049 (7)	0.0034 (6)
C43	0.0541 (8)	0.0412 (8)	0.0570 (10)	0.0079 (6)	−0.0068 (7)	0.0056 (7)
C44	0.0540 (8)	0.0378 (8)	0.0347 (7)	−0.0014 (6)	0.0069 (6)	−0.0016 (6)
C45	0.0619 (9)	0.0286 (7)	0.0445 (8)	0.0027 (6)	0.0124 (7)	−0.0061 (6)
C46	0.0535 (8)	0.0299 (7)	0.0448 (8)	0.0095 (6)	0.0098 (6)	−0.0012 (6)

### Geometric parameters (Å, °)

**Table:**

F1—C24	1.3676 (13)	C21—C26	1.3946 (16)
F2—C34	1.3610 (14)	C22—C23	1.3841 (16)
O1—C1	1.2153 (14)	C22—H22	0.9500
O2—C12	1.3660 (12)	C23—C24	1.3678 (17)
O2—C4	1.4310 (14)	C23—H23	0.9500
C1—C2	1.4770 (16)	C24—C25	1.3727 (19)
C1—C11	1.5032 (15)	C25—C26	1.3861 (17)
C2—C3	1.3288 (17)	C25—H25	0.9500
C2—H2	0.9500	C26—H26	0.9500
C3—C41	1.4625 (18)	C31—C36	1.3927 (15)
C3—H3	0.9500	C31—C32	1.3945 (17)
C4—H4A	0.9800	C32—C33	1.3837 (17)
C4—H4B	0.9800	C32—H32	0.9500
C4—H4C	0.9800	C33—C34	1.371 (2)
C5—C44	1.507 (2)	C33—H33	0.9500
C5—H5A	0.9800	C34—C35	1.367 (2)
C5—H5B	0.9800	C35—C36	1.3873 (17)
C5—H5C	0.9800	C35—H35	0.9500
C11—C12	1.4020 (16)	C36—H36	0.9500
C11—C16	1.4075 (15)	C41—C42	1.395 (2)
C12—C13	1.3869 (16)	C41—C46	1.3967 (17)
C13—C14	1.3949 (15)	C42—C43	1.380 (2)
C13—H13	0.9500	C42—H42	0.9500
C14—C15	1.3923 (15)	C43—C44	1.387 (2)
C14—C21	1.4840 (15)	C43—H43	0.9500
C15—C16	1.3938 (15)	C44—C45	1.384 (2)
C15—H15	0.9500	C45—C46	1.3850 (19)
C16—C31	1.4891 (15)	C45—H45	0.9500
C21—C22	1.3900 (17)	C46—H46	0.9500
			
C12—O2—C4	117.89 (9)	C24—C23—H23	120.9
O1—C1—C2	122.43 (11)	C22—C23—H23	120.9
O1—C1—C11	120.40 (10)	F1—C24—C23	118.13 (11)
C2—C1—C11	117.16 (10)	F1—C24—C25	118.60 (11)
C3—C2—C1	122.25 (11)	C23—C24—C25	123.27 (11)
C3—C2—H2	118.9	C24—C25—C26	117.94 (11)
C1—C2—H2	118.9	C24—C25—H25	121.0
C2—C3—C41	126.60 (12)	C26—C25—H25	121.0
C2—C3—H3	116.7	C25—C26—C21	120.86 (12)
C41—C3—H3	116.7	C25—C26—H26	119.6
O2—C4—H4A	109.5	C21—C26—H26	119.6
O2—C4—H4B	109.5	C36—C31—C32	118.37 (11)
H4A—C4—H4B	109.5	C36—C31—C16	119.36 (10)
O2—C4—H4C	109.5	C32—C31—C16	122.27 (10)
H4A—C4—H4C	109.5	C33—C32—C31	120.91 (11)
H4B—C4—H4C	109.5	C33—C32—H32	119.5
C44—C5—H5A	109.5	C31—C32—H32	119.5
C44—C5—H5B	109.5	C34—C33—C32	118.51 (13)
H5A—C5—H5B	109.5	C34—C33—H33	120.7
C44—C5—H5C	109.5	C32—C33—H33	120.7
H5A—C5—H5C	109.5	F2—C34—C35	118.54 (12)
H5B—C5—H5C	109.5	F2—C34—C33	118.68 (13)
C12—C11—C16	118.99 (10)	C35—C34—C33	122.78 (12)
C12—C11—C1	118.59 (9)	C34—C35—C36	118.27 (11)
C16—C11—C1	122.31 (10)	C34—C35—H35	120.9
O2—C12—C13	123.56 (10)	C36—C35—H35	120.9
O2—C12—C11	115.00 (10)	C35—C36—C31	121.12 (12)
C13—C12—C11	121.37 (9)	C35—C36—H36	119.4
C12—C13—C14	119.73 (10)	C31—C36—H36	119.4
C12—C13—H13	120.1	C42—C41—C46	117.55 (12)
C14—C13—H13	120.1	C42—C41—C3	122.68 (12)
C15—C14—C13	119.07 (10)	C46—C41—C3	119.77 (12)
C15—C14—C21	120.41 (9)	C43—C42—C41	120.74 (13)
C13—C14—C21	120.48 (10)	C43—C42—H42	119.6
C14—C15—C16	121.90 (10)	C41—C42—H42	119.6
C14—C15—H15	119.1	C42—C43—C44	121.73 (15)
C16—C15—H15	119.1	C42—C43—H43	119.1
C15—C16—C11	118.83 (10)	C44—C43—H43	119.1
C15—C16—C31	118.77 (9)	C45—C44—C43	117.65 (13)
C11—C16—C31	122.40 (10)	C45—C44—C5	121.74 (14)
C22—C21—C26	118.81 (11)	C43—C44—C5	120.61 (15)
C22—C21—C14	120.14 (10)	C44—C45—C46	121.26 (13)
C26—C21—C14	121.01 (11)	C44—C45—H45	119.4
C23—C22—C21	120.93 (11)	C46—C45—H45	119.4
C23—C22—H22	119.5	C45—C46—C41	121.02 (14)
C21—C22—H22	119.5	C45—C46—H46	119.5
C24—C23—C22	118.18 (12)	C41—C46—H46	119.5
			
O1—C1—C2—C3	−5.67 (19)	C22—C23—C24—C25	0.43 (17)
C11—C1—C2—C3	175.54 (11)	F1—C24—C25—C26	−179.95 (10)
C1—C2—C3—C41	179.52 (11)	C23—C24—C25—C26	0.38 (17)
O1—C1—C11—C12	123.37 (12)	C24—C25—C26—C21	−0.90 (17)
C2—C1—C11—C12	−57.82 (14)	C22—C21—C26—C25	0.60 (16)
O1—C1—C11—C16	−52.71 (16)	C14—C21—C26—C25	178.12 (10)
C2—C1—C11—C16	126.10 (11)	C15—C16—C31—C36	−42.03 (15)
C4—O2—C12—C13	−12.40 (16)	C11—C16—C31—C36	137.58 (11)
C4—O2—C12—C11	170.52 (10)	C15—C16—C31—C32	137.28 (12)
C16—C11—C12—O2	−179.66 (10)	C11—C16—C31—C32	−43.11 (16)
C1—C11—C12—O2	4.12 (15)	C36—C31—C32—C33	−2.31 (18)
C16—C11—C12—C13	3.19 (16)	C16—C31—C32—C33	178.38 (11)
C1—C11—C12—C13	−173.03 (10)	C31—C32—C33—C34	1.19 (19)
O2—C12—C13—C14	−179.30 (10)	C32—C33—C34—F2	−179.71 (12)
C11—C12—C13—C14	−2.40 (17)	C32—C33—C34—C35	0.6 (2)
C12—C13—C14—C15	−0.65 (16)	F2—C34—C35—C36	179.17 (11)
C12—C13—C14—C21	176.85 (10)	C33—C34—C35—C36	−1.1 (2)
C13—C14—C15—C16	2.93 (16)	C34—C35—C36—C31	−0.09 (18)
C21—C14—C15—C16	−174.57 (10)	C32—C31—C36—C35	1.75 (17)
C14—C15—C16—C11	−2.12 (16)	C16—C31—C36—C35	−178.91 (11)
C14—C15—C16—C31	177.50 (10)	C2—C3—C41—C42	2.5 (2)
C12—C11—C16—C15	−0.93 (15)	C2—C3—C41—C46	−177.86 (12)
C1—C11—C16—C15	175.14 (10)	C46—C41—C42—C43	2.0 (2)
C12—C11—C16—C31	179.47 (10)	C3—C41—C42—C43	−178.37 (13)
C1—C11—C16—C31	−4.47 (16)	C41—C42—C43—C44	−0.3 (2)
C15—C14—C21—C22	131.87 (11)	C42—C43—C44—C45	−1.7 (2)
C13—C14—C21—C22	−45.60 (15)	C42—C43—C44—C5	178.37 (15)
C15—C14—C21—C26	−45.62 (15)	C43—C44—C45—C46	1.9 (2)
C13—C14—C21—C26	136.91 (11)	C5—C44—C45—C46	−178.15 (13)
C26—C21—C22—C23	0.24 (16)	C44—C45—C46—C41	−0.2 (2)
C14—C21—C22—C23	−177.31 (10)	C42—C41—C46—C45	−1.7 (2)
C21—C22—C23—C24	−0.74 (16)	C3—C41—C46—C45	178.59 (12)
C22—C23—C24—F1	−179.24 (10)		

### Hydrogen-bond geometry (Å, °)

**Table:**

*D*—H···*A*	*D*—H	H···*A*	*D*···*A*	*D*—H···*A*
C4—H4B···F1^i^	0.98	2.46	3.3756 (14)	156.
C25—H25···O1^ii^	0.95	2.43	3.2812 (15)	149.

Symmetry codes: (i) *x*+1, *y*+1, *z*; (ii) *x*, *y*−1, *z*.
